# Blocking miR530 Improves Rice Resistance, Yield, and Maturity

**DOI:** 10.3389/fpls.2021.729560

**Published:** 2021-08-30

**Authors:** Yan Li, Liang-Fang Wang, Sadam Hussain Bhutto, Xiao-Rong He, Xue-Mei Yang, Xin-Hui Zhou, Xiao-Yu Lin, Aisha Anum Rajput, Guo-Bang Li, Jing-Hao Zhao, Shi-Xin Zhou, Yun-Peng Ji, Mei Pu, He Wang, Zhi-Xue Zhao, Yan-Yan Huang, Ji-Wei Zhang, Peng Qin, Jing Fan, Wen-Ming Wang

**Affiliations:** State Key Laboratory of Crop Gene Exploration and Utilization in Southwest China, Sichuan Agricultural University, Chengdu, China

**Keywords:** miR530, blast disease resistance, yield, maturity, evolution

## Abstract

MicroRNAs fine-tune plant growth and resistance against multiple biotic and abiotic stresses. The trade-off between biomass and resistance can penalize crop yield. In this study, we have shown that rice miR530 regulates blast disease resistance, yield, and growth period. While the overexpression of miR530 results in compromised blast disease resistance, reduced grain yield, and late maturity, blocking miR530 using a target mimic (MIM530) leads to enhanced resistance, increased grain yield, and early maturity. Further study revealed that the accumulation of miR530 was decreased in both leaves and panicles along with the increase of age. Such expression patterns were accordant with the enhanced resistance from seedlings to adult plants, and the grain development from panicle formation to fully-filled seeds. Divergence analysis of miR530 precursor with upstream 1,000-bp promoter sequence in 11 rice species revealed that miR530 was diverse in *Oryza sativa japonica* and *O. sativa indica* group, which was consistent with the different accumulation of miR530 in *japonica* accessions and *indica* accessions. Altogether, our results indicate that miR530 coordinates rice resistance, yield, and maturity, thus providing a potential regulatory module for breeding programs aiming to improve yield and disease resistance.

## Introduction

MicroRNAs (miRNAs) are a category of 19-24-nucleotide (nt) non-coding RNAs derived from the stem-loop of *MIR* transcripts. miRNAs regulate the expression of genes containing the reverse complementary sequences of themselves by mediating DNA methylation at the transcriptional stage, or mediating RNA cleavage at the posttranscriptional stage, or blocking protein synthesis at the translational stage (Yu et al., 2017). In plant–pathogen interaction, miRNAs act as fine tuners controlling plant immunity (Padmanabhan et al., 2009; Katiyar-Agarwal and Jin, 2010; Baldrich and San Segundo, 2016; Huang et al., 2016). miR393 is the first identified miRNA involved in plant immunity. In *Arabidopsis*, the pathogen-associated molecular pattern flg22 induces the accumulation of miR393, which positively regulates resistance and restricts the growth of bacterium *Pseudomonas syringae* by repressing the auxin signaling by downregulating the expression of the F-box auxin receptors TIR1, AFB2, and AFB3 (Navarro et al., 2006). Nowadays, a series of miRNAs have been characterized as regulators of resistance to multiple pathogens in plants and especially in the main crops, such as rice, wheat, and maize (Tang and Chu, 2017).

Rice blast is a widespread and destructive disease of cultivated rice threatening food production worldwide (Wilson and Talbot, 2009). Increasing evidence revealed that miRNAs play key roles in the regulation of rice blast disease resistance. Overexpression of miR160a leads to enhanced resistance against *Magnaporthe oryzae* accompanied by the decreased expression of three target genes encoding auxin response factors (Li et al., 2014). Overexpression of miR162 enhances rice blast resistance accompanied by the suppressed expression of *Dicer-like 1* (Salvador-Guirao et al., 2019; Li et al., 2020). Overexpression of miR166k-h positively regulates rice blast resistance through the expressions of two ethylene-insensitive 2 genes (Salvador-Guirao et al., 2018). Overexpression of miR7695 results in enhanced resistance accompanied by the decreased expression of *OsNramp6*, a negative regulator of blast disease resistance (Campo et al., 2013). Overexpression of miR398b improves rice resistance against *M. oryzae* by boosting hydrogen peroxide (H_2_O_2_) accumulation through the expression of superoxide dismutase (SOD) family genes (Li et al., 2019). Overexpression of miR812w enhances resistance by regulating the methylation of target genes (Campo et al., 2021). In contrast, miR156 (Zhang et al., 2020), miR159 (Chen et al., 2021), miR164a (Wang Z. Y. et al., 2018), miR167 (Zhao et al., 2019), miR168 (Wang et al., 2021), miR169 (Li et al., 2017), miR319b (Zhang et al., 2018), miR396 (Chandran et al., 2019), miR439 (Lu et al., 2021), and miR1873 (Zhou et al., 2020) negatively regulate rice resistance to blast fungus by suppressing the expression of their target genes.

miR530 is a conserved miRNA that exists in both monocotyledon and dicotyledon and participates in the regulation of plant development. In durum wheat, miR530 is expressed to remarkedly higher levels in leaves than that in roots (Fileccia et al., 2017). In orchardgrass (*Dactylis glomerata* L.), the expression of miR530 is variated during the vernalization and heading stage (Feng et al., 2018). In tomato, the amounts of miR530 are decreased in anthers of 7B-1, a male-sterile mutant, in comparison with that of wild type (Omidvar et al., 2015). In Siberian apricot, the expression of miR530 is gradually enhanced from 10 to 70 days after flowering in seed kernels; conversely, the target gene *P2C37* is reversely expressed and responsive to abscisic acid (ABA) (Niu et al., 2016). Except for the participation in plant growth, miR530 is also responsive to environmental stresses. In *Caragana korshinskii*, the amounts of miR530 are significantly decreased in water stress conditions (Ning et al., 2017). In grapevine berry, the amounts of miR530 are increased associated with suppressed mRNA levels of a target gene containing the Plus-3 domain under the high-fluence rates of UV-B (Sunitha et al., 2019). In flax (*Linum usitatissimum*), miR530 is up-regulated under alkaline or alkaline salt stress, whereas it is down-regulated under salt stress (Yu et al., 2016).

Besides, miR530 is also involved in the regulation of the synthesis of secondary metabolites and the responses to the biotic stresses. In *Withania somnifera*, miR530 regulates the biosynthesis of withanolide, a secondary metabolite used as one of the most important medicines in India (Srivastava et al., 2018). In *Persicaria minor* (kesum), miR530 is highly enhanced by pathogenic fungus *Fusarium oxysporum*, which acts as an inducer of terpenoid biosynthesis in kesum, suggesting the involvement of miR530 in terpenoid biosynthesis (Samad et al., 2019). In chickpea (*Cicer arietinum*), the amounts of miR530 are increased in response to salt and highly up-regulated by wilt infection caused by the fungus *F. oxysporum* f.sp. *ciceris* (Kohli et al., 2014). In maize, miR530 is up-regulated by fungus *Exserohilum turcicum* (Pass.), the agent of northern leaf blight (Wu et al., 2014). These reports indicate that miR530 participates in plant resistance against multiple pathogens.

In rice, the accumulation of miR530 is decreased under N starvation, suggesting a potential role in rice nutrient homeostasis (Cai et al., 2012). Overexpression of miR530 results in yield loss accompanied by decreased grain size and panicle branching, whereas blocking miR530 leads to increased grain yield (Sun et al., 2020). Further study reveals that the expression of *MIR530* is controlled by *Phytochrome-Interacting factor-like gene 15* (*OsPIL15*), which directly binds to the G-box elements in the promoter of *MIR530* (Sun et al., 2020). However, the roles of miR530 in rice resistance remain elusive.

In this study, to explore the roles of miR530 in rice development and immunity, we constructed the transgenic lines overexpressing miR530 and silencing miR530, respectively. We examined the blast disease resistance, growth period, and yield traits of these transgenic lines. We also analyzed the expression pattern of miR530 throughout the growth period and the evolution of the *MIR530* gene in rice species. Our results characterized an miRNA that could be exploited to improve immunity, yield, and maturity simultaneously in rice.

## Materials and Methods

### Plant Materials and Growth Conditions

The rice (*Oryza sativa* L.) accessions Kasalath (ssp. *indica*), 9311 (ssp. *indica*), IR64 (ssp. *indica*), Lijiangxin Tuan Heigu (LTH, ssp. *japonica*), International Rice Blast Line Pyricularia-Kanto51-m-Tsuyuake (IRBLkm-Ts, ssp. *japonica*), Zhonghua11 (ZH11, ssp. *japonica*), and Nipponbare (ssp. *japonica*) were used in this study. For resistance assay, the rice plants were grown in a greenhouse with a 28/23 ± 1°C day/night temperature, 70% relative humidity, and a 14/10-h light/dark period. For yield trait assay, the rice plants were grown in a paddy field in Wenjiang District, Chengdu, China (36°N, 103°E) during the rice-growing season from mid-April to late-September in 2019 and 2020.

### Plasmid Construction and Genetic Transformation

The transgenic lines were generated following previous protocols (Li et al., 2017). To construct the transgenic lines overexpressing miR530, the sequence of the *MIR530* gene containing 400-bp upstream and 318-bp downstream sequences was amplified from total genomic DNA of Kasalath with primers miR530-F and miR530-R (Supplementary Table 1). We cloned the amplified fragment in binary vector 35S-pCAMBIA1300 and obtained the construct *p35S:MIR530* overexpressing miR530. To construct the target mimic of miR530 (MIM530), we constructed the target mimic sequences of miR530 (TCCACGTCTCCACAGTCTACGTTG) containing the cutting sites of restrictive enzymes by annealing with primers MIM530-*Bam*HI-F and MIM530-*Bgl*II-R (Supplementary Table 1). Then, the annealed double-strand fragment was inserted into the *Arabidopsis IPS1* gene to substitute the target site of miR399 at *Bam*HI and *Bgl*II sites as described previously (Franco-Zorrilla et al., 2007; Li et al., 2017). We cloned the reconstructed *IPS1*-MIM530 fragment into the binary vector pCAMBIA1300 and obtained the construct *p35S:MIM530* overexpressing the target mimic of miR530. Then, the vectors *p35S:MIR530* and *p35S:MIM530* were transformed into the background variety Kasalath by *Agrobacterium* strain EHA105, respectively, to acquire the transgenic lines OX530 and MIM530. The positive transgenic lines were screened with Hygromycin B. The accumulation of miR530 in OX530 and MIM530 transgenic lines was examined following a previous report (Li et al., 2020).

### Trait Measurements

The agronomic traits, such as plant height, panicle number per plant, grain number per panicle, seed setting rate, 1,000-grain weight, grain yield per plant, seed length, and seed width were measured from five plants growing in the middle of the three rows in the paddy yard. The seeds were harvested at the full-mature stage and dried in a 42°C oven for 1 week. Then, the dried seeds were used to measure the yield traits using a grain analysis system (SC-A, Wanshen Ltd., Hangzhou, China). All the data were analyzed by a one-way ANOVA followed by *post hoc* Tukey’s honestly significant difference (HSD) analysis with significant differences (*P* < 0.05).

### RNA Extraction and Gene Expression Analyses

Reverse-transcription quantitative PCR (RT-qPCR) analyses were carried out to examine the expression of *MIR530* and the indicated genes. Total RNAs were extracted from rice leaves using TRIzol reagent (Thermo Fisher Scientific, Shanghai, China) following the instruction of the manufacturer. The accumulation of miR530 was examined in T1 plants. To determine the amounts of miR530, total RNA was reverse-transcribed using an miRNA-specific stem-loop RT primer (Supplementary Table 1) with the PrimeScript^TM^ RT Reagent Kit with gDNA Eraser (Takara Biotechnology, Japan), and the RT product was subsequently used as a template for quantitative PCR (qPCR) by using the miRNA-specific forward primer and the universal reverse primer (Supplementary Table 1). Small nuclearRNA (snRNA) U6 was used as an internal reference to normalize the relative amounts of miR530. qPCR was performed using specific primers and SYBR Green mix (QuantiNova SYBR Green PCR Kit, QIAGEN, China) with three technical replicates by BIO-RAD C1000TM Thermal Cycler (Bio-Rad Inc., China). The 2^–ΔΔCT^ method was exploited to analyze the relative expression levels of miRNAs. Kasalath was used as a control to normalize the relative levels of miR530. To detect the expression of genes, the first-strand cDNA was synthesized from 1 μg of total RNA using PrimeScript^TM^ RT Reagent Kit with gDNA Eraser (TaKaRa Biotechnology, Dalian, China) according to the instruction of the manufacturer. RT-qPCR was performed using specific primers (Supplementary Table 1) and SYBR Green mix (QuantiNova SYBR Green PCR Kit, QIAGEN, China) with BIO-RAD C1000TM Thermal Cycler (Bio-Rad Inc., China). The rice ubiquitin (*UBQ*) gene was used as an internal reference to normalize the relative expression levels of genes.

### Pathogen Infection Analysis

*Magnaporthe oryzae* strains Guy11, 97-27-2, NC-10, NC-34, CRB1, and GZ8 were used for resistance and defense response assays. 97-27-2 and CRB1 are strains isolated from rice fields in North China, and GZ8 is another green fluorescent protein-tagged strain Zhong8-10-14 isolated from a rice field in North China. NC-10 and NC-34 were the strains derived from a paddy yard in Sichuan province, China. The strains were cultured in plates containing oatmeal tomato agar medium at 28°C for 2 weeks with the 12/12-h light/dark cycles. After getting rid of the surface mycelia with distilled water, the plates were further incubated for 3 days with consistent light treatment to promote sporulation. Then, the spores were collected with distilled water, and the concentration of the inoculums of the spores was diluted to 1 × 10^5^ or 5 × 10^5^ conidia ml^–1^ for inoculation. For resistance assay, punch- or spray inoculation was carried out following a previous report (Kong et al., 2012). In brief, conidia suspension (5 × 10^5^ conidia ml^–1^) of indicated strains was punch-inoculated at the wounded sites or spray-inoculated on the three- to five-leaf-stage seedlings. Lesion formation was examined at 4–6 days postinoculation. The fungal biomass was determined by using the DNA amounts of fungal *Mopot2* against rice DNA amounts of ubiquitin through qPCR (Li et al., 2017).

### Hydrogen Peroxide Accumulation Assay

To observe the H_2_O_2_ accumulation in rice plants, the leaves or the 5-cm-long leaf sheaths of the three- to five-leaf-stage seedlings were inoculated with *M*. *oryzae* strain Guy11 at the concentration of 5 × 10^5^ conidia ml^–1^ as described previously (Kankanala et al., 2007). Then, the inoculated leaves or the excised epidermal layer of the leaf sheaths were incubated in 1 mg/ml 3,3′-diaminobenzidine (DAB; Sigma–Aldrich, Germany) at 22°C for 8 h. The DAB-stained leaves and leaf sheaths were cleaned in 95% ethanol and then observed under a microscope (Zeiss Imager A2, Carl Zeiss, Germany).

### Genetic Diversity Analysis

We analyzed the single-nucleotide polymorphisms of miR530 precursor with the 1,000-bp upstream sequence in 11 wild rice species (Gramene Database, http://www.gramene.org/) using MEGA5.05 software^1^ to characterize the evolution of genetic variation of *MIR530* locus in rice species.

## Results

### miR530 Is Responsive to *M. oryzae*

**FIGURE 1:**
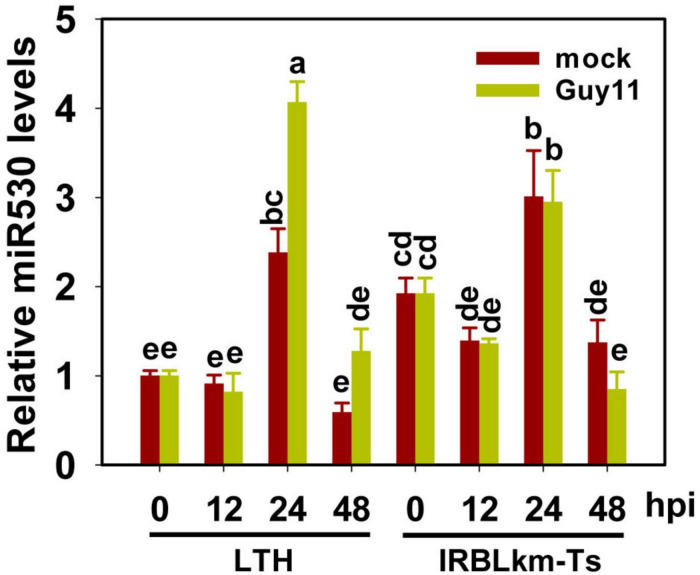
miR530 is differentially responsive to blast fungus in susceptible and resistant accessions. The data of reverse-transcription quantitative PCR (RT-qPCR) show miR530 levels in Lijiangxin Tuan Heigu (LTH) and International Rice Blast Line Pyricularia-Kanto51-m-Tsuyuake (IRBLKm-Ts) with or without *Magnaporthe oryzae* treatment. The data are shown as mean ± SD (*n* = 3 independent samples). The mRNA levels were normalized to that of the mock sample of LTH at 0 h postinoculation. Different letters above the bars indicate significant differences (*P* < 0.01) as determined by the one-way ANOVA analysis.

In rice, only one *MIR530* gene was identified locating on Chromosome 4^2^. We first explored whether miR530 is involved in rice blast disease resistance. We examined the amounts of miR530 in a susceptible rice accession LTH and a resistant accession IRBLkm-Ts following the infection of *M. oryzae* strain Guy11. The accumulation of miR530 was significantly increased in LTH at 24 and 48 h postinoculation (hpi) in comparison with the mock samples treated with H_2_O_2_, whereas that was slightly fluctuated in IRBLkm-Ts (Figure 1), suggesting that miR530 was involved and possibly played a negative role in rice resistance against *M. oryzae*.

### Blocking miR530 Enhances Rice Blast Disease Resistance

**FIGURE 2:**
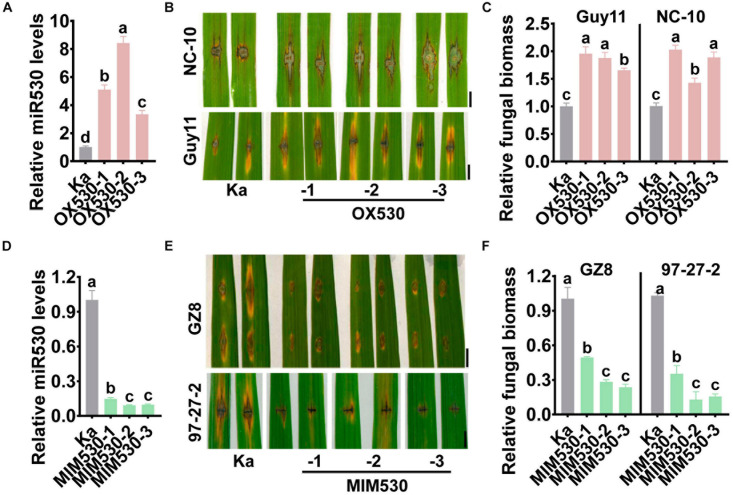
Blocking miR530 enhances rice blast disease resistance. **(A,D)** The relative amounts of mature miR530 in the Kasalath control (Ka), the transgenic lines overexpressing *MIR530* gene (OX530), or the transgenic lines expressing a target mimic of miR530 (MIM530). The reverse transcription (RT) was carried out with total RNA and miR530-specific stem-loop RT primer (Supplementary Table 1). The RT product was subsequently used as a template for qPCR to detect the amounts of miR530. The amounts of snRNA U6 were detected and used as an internal reference. **(B,E)** The blast disease phenotypes on leaves of the Kasalath control and the transgenic lines overexpressing miR530 (OX530; **B**), or the transgenic lines expressing a target mimic of miR530 (MIM530; **E**) at 5 days postinoculation of the indicated *Magnaporthe oryzae* strains by punch inoculation, respectively. Bar = 5 mm. **(C,F)** The relative fungal biomass of indicated strains on the Kasalath control and OX530 **(B)**, or MIM530 **(E)**. The fungal biomass in **(C,F)** was determined by using the ratio of DNA levels of *M. oryzae MoPot2* against that of the rice *ubiquitin* gene. The data are shown as mean ± SD (*n* = 3 independent samples). Different letters above the bars indicate significant differences (*P* < 0.01) as determined by the one-way ANOVA analysis. Similar results were obtained in at least two independent experiments.

To further investigate the roles of miR530 in rice blast disease resistance, we constructed the transgenic lines overexpressing the *MIR530* gene (OX530) and expressing a target mimic of miR530 (MIM530) to block miR530 from binding with target genes (MIM530, Supplementary Figure 1), respectively. The OX530 lines displayed significantly increased accumulation of miR530 in comparison with the Kasalath control (Ka; Figure 2A), whereas the MIM530 lines exhibited significantly reduced amounts of miR530 (Figure 2D). We detected the resistance of OX530 and MIM530 following punch- and spray inoculation of *M. oryzae* strains. OX530 plants exhibited compromised resistance to the *M*. *oryzae* strains Guy11, NC-10, and NC-34 with obviously more and larger disease lesions and supported more fungal growth (Figures 2B,C and Supplementary Figures 2A,B). Conversely, MIM530 exhibited elevated resistance to strains GZ8, 97-27-2, and NC-10 accompanied by smaller disease lesions and less fungal biomass than the Kasalath control (Figures 2E,F and Supplementary Figures 2C,D). Moreover, MIM530 lines also showed enhanced resistance to multiple *M. oryzae* strains derived from North China (Wang W. W. et al., 2018) and South China (Supplementary Figures 3A,B). These results demonstrated that miR530 negatively regulated rice blast disease resistance, and blocking miR530 could improve the resistance.

### Blocking miR530 Enhances Rice Defense Responses Against *M. oryzae*

**FIGURE 3:**
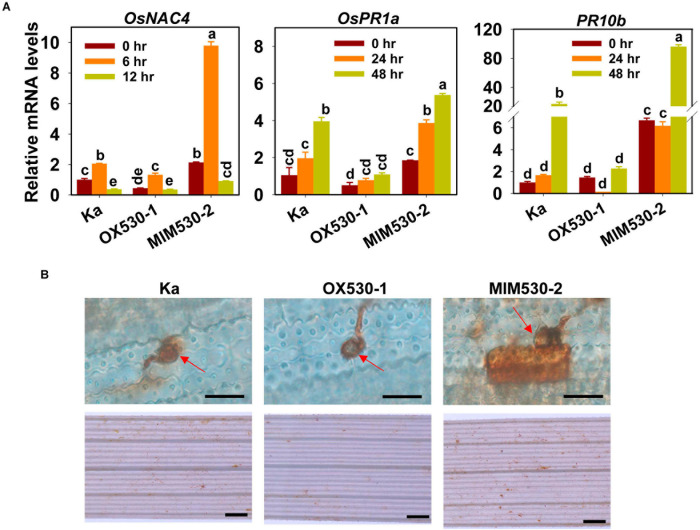
miR530 regulates *Magnaporthe oryzae*-induced defense responses. **(A)** The relative mRNA levels of the defense-related genes (*OsNAC4*, *OsPR1A*, and *OsPR10b*) in the Kasalath controls (Ka) and the indicated lines at the indicated time points. The data are shown as mean ± SD (*n* = 3 independent samples). The mRNA levels were normalized to that in the indicated control. Different letters above the bars indicate significant differences (*P* < 0.01) as determined by the one-way ANOVA analysis. **(B)** The hydrogen peroxide (H_2_O_2_) accumulation in the Kasalath controls and indicated lines at 48 h postinoculation of *M. oryzae* strain Guy11. The accumulation of H_2_O_2_ was stained by 3,3′-diaminobenzidine (DAB), and the intensity of brown indicates the amounts of H_2_O_2_. The red arrows indicate appressoria formed from conidia. The photographs given above were taken with the leaves using a microscope (Zeiss imager A2, Germany). Scale bars = 10 μm. The photographs given below were taken with the leaves with a stereomicroscope (Zeiss imager A2, Germany). Scale bars = 50 μm. These experiments were repeated two times with similar results.

We then examined the expression of defense response-related genes on the treatment of *M. oryzae*, including *OsNAC4* (for *O. sativa* no apical meristem, *Arabidopsis* transcription activation factor, and *cup-shaped cotyledon* domain transcription factor4), *pathogenesis-related protein1a* (*PR1a*), and *PR10b*. *OsNAC4* was an early responsive gene induced by *M. oryzae* (Park et al., 2012), the expression of which was induced significantly at 6 hpi and was suppressed at 12 hpi. In contrast, *PR1a* and *PR10b* were late-responsive genes, the expression of which was triggered at 24 and 48 hpi (Figure 3A). Moreover, consistent with the resistance phenotype, the mRNA levels of the three genes were constitutively enhanced in MIM530 but decreased in OX530 in comparison with those in the Kasalath control (Figure 3A). In rice, H_2_O_2_ accumulation is another typical defense response triggered by *M. oryzae*. We detected the H_2_O_2_ production in OX530 and MIM530. The DAB-stained intensity indicated H_2_O_2_ accumulation. H_2_O_2_ was highly accumulated in the local invasive cell in MIM530 in comparison with that of the Kasalath control at 48 hpi, whereas less amount of H_2_O_2_ was accumulated in the invaded cells of OX530 (Figure 3B). These results demonstrated that blocking miR530 could boost rice defense responses against *M. oryzae*.

### Blocking miR530 Shortens the Rice Growth Period

**FIGURE 4:**
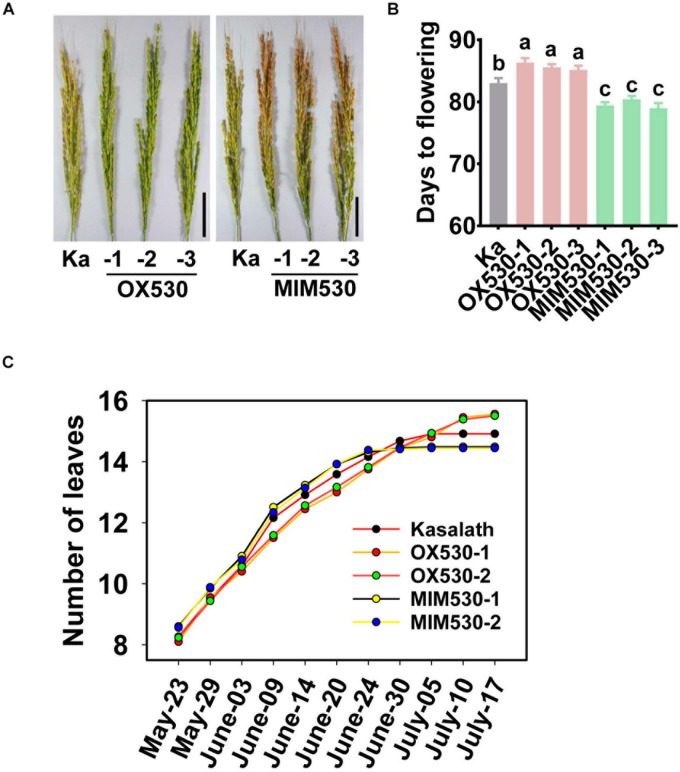
Blocking miR530 shortens rice flowering time. **(A)** The panicle morphology of the Kasalath control (Ka), OX530, and MIM530. Bars = 5 cm. **(B)** The flowering time of the Kasalath control, OX530, and MIM530 planted in paddy yard in Wenjiang District, Chengdu City, Sichuan Province, China during the regular season from April to September in 2019. The data are shown as mean ± SD (*n* = 10 independent plants). Different letters above the bars indicate significant differences (*P* < 0.05) as determined by the one-way ANOVA analysis. **(C)** Quantification of leaf number per main tiller of the Kasalath control, OX530, and MIM530 lines. The data are shown as mean ± SD (*n* = 10 independent plants).

Except for the regulation of rice resistance, miR530 was also involved in the regulation of rice growth period and maturity. OX530 displayed an approximate 3-days-later flowering time and late maturity in comparison with the control, whereas MIM530 showed a 3-days-earlier flowering time and early maturity when planted in a paddy yard in the Sichuan Basin, South of China during the normal rice growth period from April to September (Figures 4A,B). Further study revealed that OX530 developed 15–16 leaves on average, whereas MIM530 developed approximately 14 leaves in comparison with the control developing 15 leaves (Figure 4C), indicating a longer vegetative growth period in OX530 and a shorter period in MIM530. These results showed that miR530 controlled rice growth period and maturity, and blocking miR530 could shorten rice flowering and ripeness.

### Blocking miR530 Enhances Grain Yield

**FIGURE 5:**
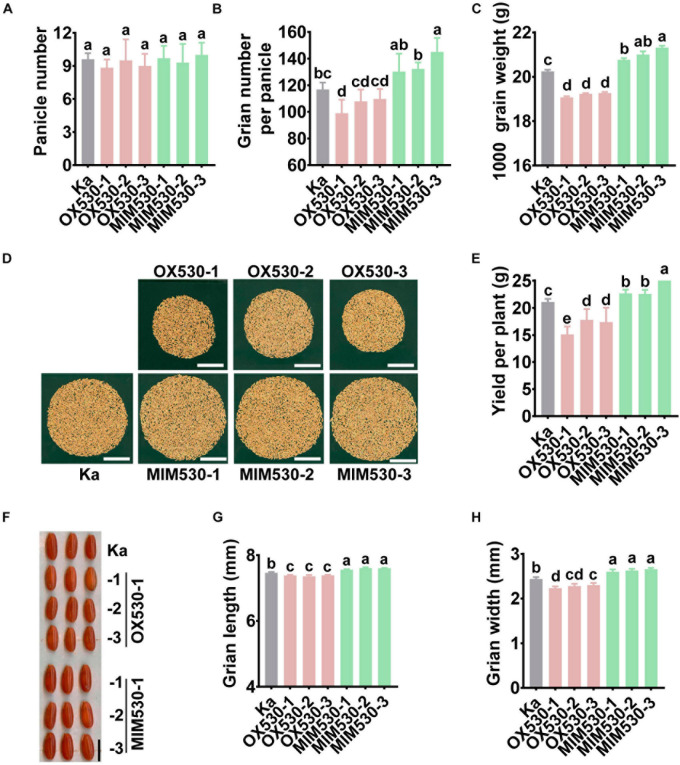
Blocking miR530 enhances grain yield. **(A–C,E,G,H)** The panicle number per plant **(A)**, grain number per panicle **(B)**, grain weight **(C)**, yield per plant **(E)**, seed length **(G)**, and seed width **(H)** of the Kasalath control (Ka), OX530, and MIM530 planted in paddy yard in Wenjiang District, Chengdu City, Sichuan Province, China during the regular season from April to September in 2019. The data are shown as mean ± SD (*n* = 10 independent plants). Different letters above the bars indicate significant differences (*P* < 0.05) as determined by the one-way ANOVA analysis. **(D)** The photograph of grains per plant of the Kasalath control, OX530, and MIM530. Scale bars = 5 cm. **(F)** The husked seed morphology (**(F)**, scale bars = 5 mm) of the Kasalath control, OX530, and MIM530.

The alteration in the amounts of miR530 led to changed agronomic traits in the Nipponbare background (Sun et al., 2020). Consistent with the results in Nipponbare, OX530 lines in the Kasalath background exhibited shorter plants, whereas MIM530 showed higher plants (Supplementary Figures 4A,B and Supplementary Table 2). Rice yield was determined by three key agronomic traits, such as panicle number, grain number per panicle, and grain weight. In this study, we examined the effect of miR530 on the yield-related agronomic traits in the Kasalath background. Both OX530 and MIM530 showed an unchanged panicle number in comparison with the Kasalath control (Figure 5A and Supplementary Table 2). Consistent with the results in Nipponbare, OX530 lines in the Kasalath background exhibited less grain yield per plant due to fewer filled seeds per panicle and smaller seeds than the Kasalath control (Figures 5B–F and Supplementary Table 2). Conversely, MIM530 displayed more grain yield resulted from bigger seeds, more filled grains per panicle (Figures 5B–F and Supplementary Table 2). These results demonstrated that miR530 negatively regulates rice grain yield, and blocking miR530 could improve grain yield. Intriguingly, both OX530 and MIM530 exhibited a slight but not significant decrease of seed setting rate (SSR) in comparison with the Kasalath control (Supplementary Table 2).

### miR530 Coordinates Rice Resistance, Yield, and Maturity Through Spatiotemporally Altered Expressions

**FIGURE 6:**
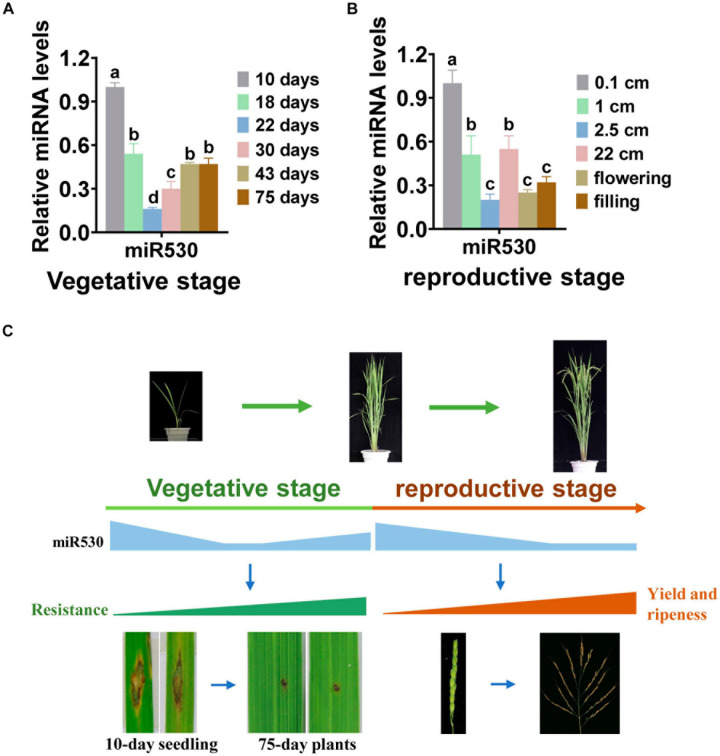
miR530 dynamically expressed during the whole growth period in rice. **(A)** The accumulation of miR530 in rice leaves during the vegetative stage. **(B)** The accumulation of miR530 in rice panicles during the reproductive stage. For **(A,B)**, the data are shown as mean ± SD (*n* = 3 independent samples). The mRNA levels were normalized to that in the indicated control. Different letters above the bars indicate significant differences (*P* < 0.01) as determined by the one-way ANOVA analysis. **(C)** A model shows the relationship among the amount of miR530, resistance, yield, and growth period throughout the growth period. During the whole growth period, miR530 is gradually decreased to coordinate disease resistance, maturity, and panicle development. The photograph shows the disease symptom that was captured from 10-day seedlings and 75-day plants inoculated with GZ8.

Our results showed that blocking miR530 resulted in improved resistance, ripeness, and yield traits. To learn about how miR530 coordinates these different traits, we performed a time course examination of the expression of miR530 in rice leaves and panicles throughout the whole growth period. Intriguingly, the amounts of miR530 were significantly reduced in leaves of 18-day-old plants in comparison with that in 10-day-old seedlings, as well as reduced to the lowest levels in 22-day-old plants, but slightly increased to the level of 18-day-old plants during the later vegetative stage (Figure 6A). Similarly, the expression of miR530 in panicles was decreased significantly from the early panicle-formation stage to the grain-filling stage in comparison with that in 0.1-cm-long panicles during the reproductive stage (Figure 6B). The gradually decreased expression of miR530 was reversely consistent with the enhanced plant adult resistance, which enhanced gradually when the plants grow up (Figure 6C). Moreover, the decreased amounts of miR530 were helpful to improve yield and accelerate ripeness at the reproductive stage (Figure 6C). Thus, the dynamic expression of miR530 may coordinate rice yield traits, growth period, and resistance.

### The Genotype of *MIR530* Is Diverse in *Oryzae* Species

**FIGURE 7:**
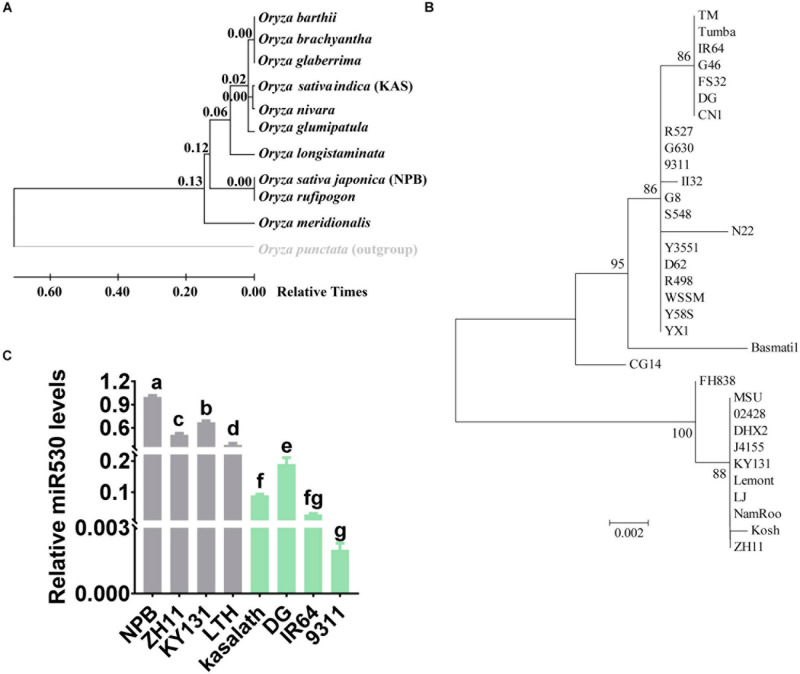
MIR530 was diverse in rice species. **(A)** Phylogenetic tree inferred by analyzing the sequences of *MIR530* gene (miR530 precursor with 1,000-bp upstream promoter sequence) in 11 wild rice species using the neighbor-joining method. The numbers at the nodes are relative divergence times. **(B)** Phylogenetic tree inferred by analyzing the *MIR530* gene in 32 *O. sativa* accessions covering all known subpopulations and an *Oryza glaberrima* accession. The numbers at the nodes are relative divergence times. **(C)** The relative amount of mature miR530 in the indicated *O. sativa japonica* and *O. sativa indica* accessions. The RT was carried out with total RNA and miR530-specific stem-loop RT primer (Supplementary Table 1). The RT product was subsequently used as a template for qPCR to detect the amounts of miR530. The amounts of snRNA U6 were detected and used as an internal reference.

miR530 is correlated with rice yield, maturity, and resistance, suggesting miR530 acts as a key regulator in rice growth and resistance. We then analyzed the evolution of the *MIR530* (miR530 precursor with 1,000-bp upstream promoter sequence) in 11 *Oryza* species. We found that *O. punctata*, a wild rice species, was an out-group (Figure 7A). Intriguingly, MIR530 was conserved in *O. brachyantha*, the most primitive of the surveyed wild species, suggesting that the *MIR530* locus may be generated during the formation of wild *Oryza* species and evolved in different species. Surprisingly, miR530 was diverse in the *japonica* subspecies and *indica* subspecies (Figure 7A). We then analyzed the evolution of *MIR530* in the 32 *O. sativa* accessions covering all known subpopulations including nine *japonica* accessions, 21 *indica* accessions, N22 [*centrum-Aus* (cA)], and Basmati-1 [*centrum-Basmati* (cB)]. CG14 is an *O. glaberrima* accession defined as an ancestral species and used as a control (Supplementary Figure 5; Qin et al., 2021). The nine *japonica* accessions and the *indica* accession J4155 were clustered together; the 21 *indica* accessions and the cA accession N22 were clustered together; the cB accession Basmati-1 was an out-group from the *indica* group (Figure 7B and Supplementary Figure 5). CG14 was an out-group from both *japonica* and *indica* accessions; however, it was closer to the *indica* group than to the *japonica* group (Figure 7B and Supplementary Figure 5). These data indicated that *MIR530* was existed globally in the *O. sativa* accessions covering all known subpopulations, and first occurred in *indica* subspecies, then evolved in *japonica* subspecies. We then examined the accumulation of miR530 in *japonica* accessions and *indica* accessions used globally in rice production. In accordance with the diversity, the accumulation of miR530 in the *japonica* accessions (Nipponbare, Zhonghua11, KY131, and LTH) was significantly higher than those in the *indica* accessions (Kasalath, Digu, IR64, and 9311; Figure 7C), indicating that the expression of miR530 was differentially regulated in different subspecies.

## Discussion

In this study, we demonstrated that miR530 negatively regulates rice blast disease resistance. Blocking miR530 by overexpressing a target mimic of miR530 enhanced rice resistance and defense responses (Figures 2, 3). However, how miR530 compromised the resistance is unknown. It was reported that miR530 targets *LOC_Os05g34720* (Zheng et al., 2012), which encodes a bifunctional dihydrofolate reductase/thymidylate synthase (DHFR-TS; http://rice.plantbiology.msu.edu/). DHFR-TS participates in the maintenance of the redox balance of many proteins by contributing to the production of nicotinamide adenine dinucleotide phosphate (NADPH), which is the subtract of NADPH oxidase and provides the electrons to O⋅_2_−, i.e., the reactive oxygen that could be converted into H_2_O_2_ by SOD (Gorelova et al., 2017). Therefore, whether miR530 controls H_2_O_2_ accumulation by regulating the expression of *LOC_Os05g34720* could be an interesting focus of research.

We also demonstrated that miR530 compromised grain yield. Blocking miR530 enhanced yield accompanied by increased grain number per panicle and grain weight (Figure 5). A previous study has revealed that miR530 targeted *OsPL3*, which encodes a Plus-3 domain-containing protein, to regulate rice yield (Sun et al., 2020). The mutants of *OsPL3* and OX530 lines showed compromised grain yield resulting from reduced grain size and panicle branch (Sun et al., 2020), indicating that miR530 suppressed rice grain yield by altering the grain size and panicle architecture by *OsPL3*. In this study, we confirmed that miR530 acted as a negative regulator of rice grain yield in the Kasalath background, and blocking miR530 enhanced rice yield (Figure 5). However, whether *OsPL3* is involved in miR530-regulated rice maturity and resistance is unknown and needs further study.

Except for the involvement in rice resistance and yield, miR530 also regulates the rice growth period. Blocking miR530 shortened the flowering time leading to earlier flowering and seed maturity (Figure 4). Conversely, overexpressing miR530 prolonged the growth period and delayed seed maturity (Figure 4). Consistently, miR530 was dynamically decreased in panicles during the reproductive stage. The gradual decrease in miR530 was reversely correlated with the maturity of panicles. It will be helpful for learning the underlined regulating network of miR530 by identifying the downstream target genes involving in the regulation of maturity.

miR530 existed globally in both monocotyledon and dicotyledonous^3^ and was participated in multiple development and responses to environmental stresses. However, miR530 was not identified in *Arabidopsis* and maize, indicating a unique function during plant divergence and speciation. In this study, the analysis of the genetic variations suggested that *MIR530* might generate during the formation of wild *Oryza* species. A previous report also revealed that two insertion–deletion polymorphisms were identified in the promoter sequences of the *indica* and *japonica* varieties, suggesting that the *MIR530* locus was artificially selected during rice domestication and acted as a key factor in rice development (Sun et al., 2020). In this study, we showed that these polymorphisms in *MIR530* existed in many rice species, and the *indica* subspecies were closer to the ancestral species (Figure 7). Consistently, the accumulation of miR530 was different in the examined *japonica* and *indica* cultivars: the relative amounts of miR530 were lower in the examined *indica* accessions than those in the examined *japonica* accessions. These results suggested a correlation between the genetic variations and the miR530 accumulation. Therefore, it is necessary to study the upstream signaling pathway of miR530 and to identify the potential genes that are useful to improve yield and disease resistance simultaneously.

## Data Availability Statement

The original contributions presented in the study are included in the article/Supplementary Material, further inquiries can be directed to the corresponding author/s.

## Author Contributions

YL and W-MW conceived the experiment. YL, W-MW, L-FW, SB, X-RH, X-MY, X-HZ, and AAR carried out the experiment. G-BL, J-HZ, HW, Z-XZ, J-WZ, JF, Y-YH, and PQ analyzed the data. Y-PJ, S-XZ, and MP carried out the field trial. YL and W-MW wrote the manuscript. All authors contributed to the article and approved the submitted version.

## Conflict of Interest

The authors declare that the research was conducted in the absence of any commercial or financial relationships that could be construed as a potential conflict of interest.

## Publisher’s Note

All claims expressed in this article are solely those of the authors and do not necessarily represent those of their affiliated organizations, or those of the publisher, the editors and the reviewers. Any product that may be evaluated in this article, or claim that may be made by its manufacturer, is not guaranteed or endorsed by the publisher.

## Footnotes

1
http://www.megasoftware.net/

2
www.mirbase.com

3
http://www.pmiren.com/search

## Supplementary Material

Supplementary Figure 1 The sequences and alignment of miR530 and MIM530. *MIR530* gene was located at Chromosome 4. The unmatched nucleotide in MIM530 is shown in red.

Supplementary Figure 2 miR530 regulates rice resistance against blast fungus. **(A,C)** Disease phenotypes on leaves of the Kasalath control (Ka), OX530, and MIM530 lines following spray inoculation with the indicated *Magnaporthe oryzae* strains. Scale bar = 1 cm. **(B,D)** The quantification analysis of the fungal biomass in **(A,C)**. The relative fungal biomass was measured by using the ratio of DNA levels of *Pot2* genes of *M. oryzae* against the rice genomic ubiquitin DNA levels. Similar results were obtained in at least two independent experiments.

Supplementary Figure 3 Blocking miR530 enhances rice resistance to multiple *Magnaporthe oryzae* strains. **(A,B)** The disease phenotypes on leaves of the Kasalath control (Ka) and MIM530 following punch inoculation of field-derived *M. oryzae* strains from South China **(A)** and North China **(B)**. The phenotype was captured at 5 days postinoculation. Scale bars = 5 mm. Similar results were obtained in at least two independent experiments.

Supplementary Figure 4 miR530 regulates plant height. **(A)** Photographs of the gross morphology of the Kasalath control (Ka), OX530, and MIM530 lines planted in paddy yard in Wenjiang District, Chengdu City, Sichuan Province, China during the regular season from April to September in 2019. Scale bars = 50 cm. **(B)** The plant height of the indicated lines in **(A)**. The data are shown as mean ± SD (*n* = 10 independent plants). Different letters above the bars indicate a significant difference (*P* < 0.05) as determined by the one-way ANOVA analysis.

Supplementary Table 1 Primers used in this study.

Supplementary Table 2 Agronomic traits of the Kasalath control and the transgenic lines.
