# Supplementary material for: Blocking miR530 Improves Rice Resistance, Yield, and Maturity
Source: Front Plant Sci. 2021 Aug 30;12:729560. doi: 10.3389/fpls.2021.729560 (PMC8435866; doi:10.3389/fpls.2021.729560)
Supplement: Supplementary Figure 1 — The sequences and alignment of miR530 and MIM530. MIR530 gene was located at Chromosome 4. The unmatched nucleotide in MIM530 is shown in red. [file Data_Sheet_1.PDF]

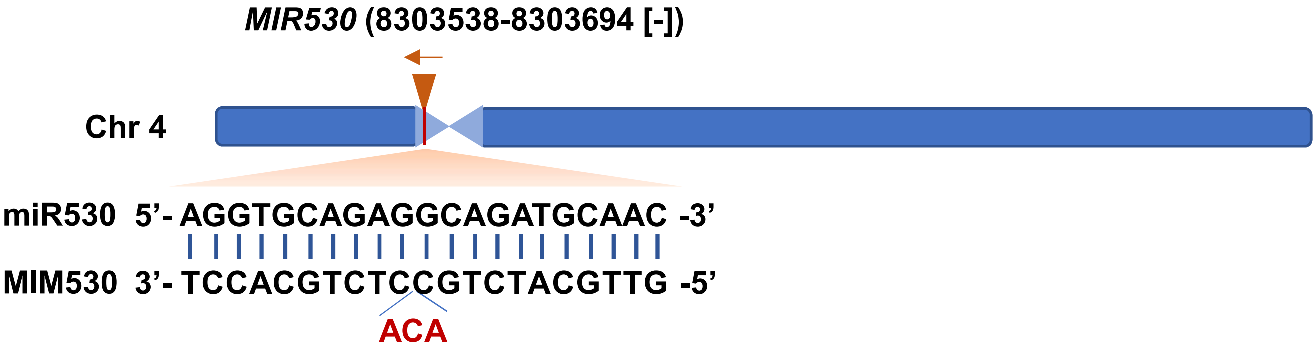

Figure S1 The sequences and alignment between miR530 and MIM530. *MIR530* gene was located at Chromosome 4. The unmatched nucleotide in MIM530 is shown in red.

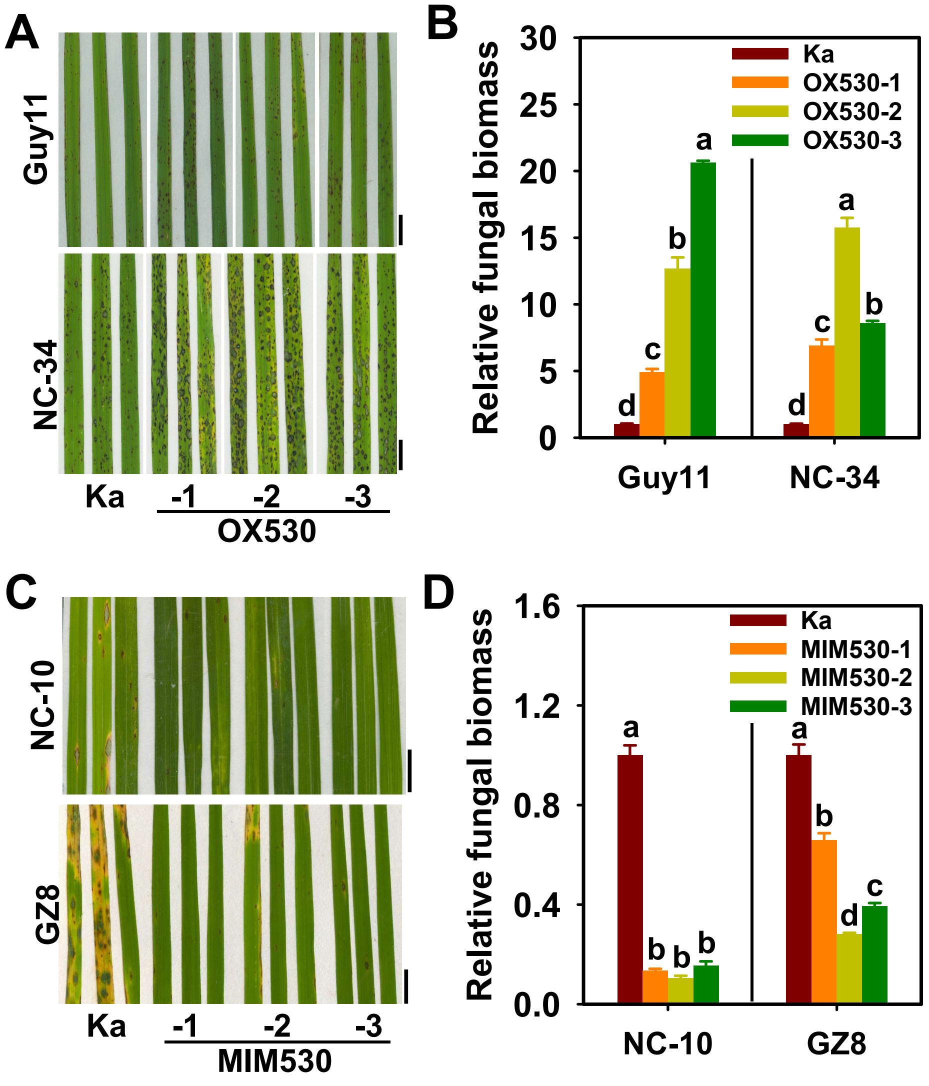

Figure S2 miR530 regulates rice resistance against blast fungus. (A and C) Disease phenotypes on leaves of the Kasalath control, OX530, and MIM530 lines following spray-inoculation with the indicated *Magnaporthe oryzae* strains. Scale bar = 1 cm. (B and D) Quantification analysis of the fungal biomass in (A) and (C). The relative fungal biomass was measured by using the ratio of DNA levels of *M. oryzae Pot2* genes against the rice genomic ubiquitin DNA levels. Similar results were obtained in at least two independent experiments.

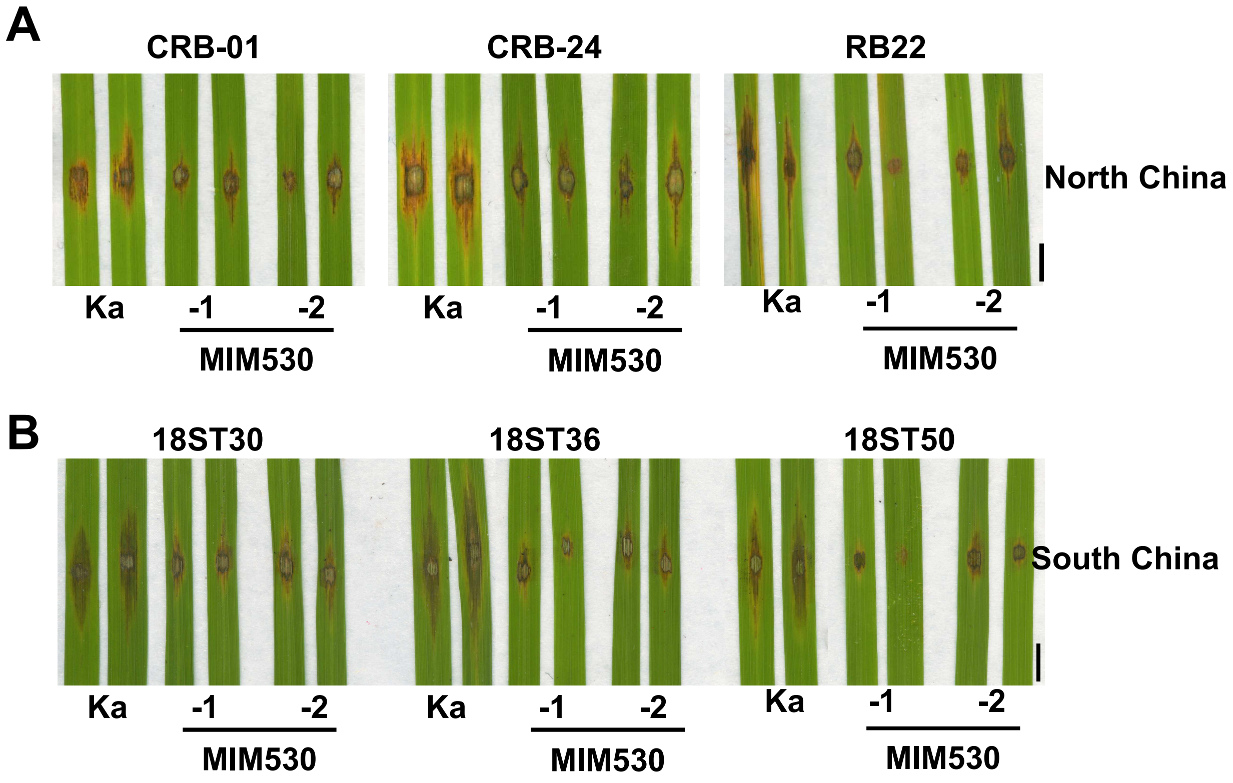

Figure S3 Blocking miR530 enhances rice resistance to multiple *Magnaporthe oryzae* strains. (**A** and **B**) The Disease phenotypes on leaves of the Kasalath control (Ka) and MIM530 following punch-inoculation of field-derived *M. oryzae* strains from South China (A) and North China (B). The phenotype was captured at five days post-inoculation. Scale bars = 5mm. Similar results were obtained in at least two independent experiments.

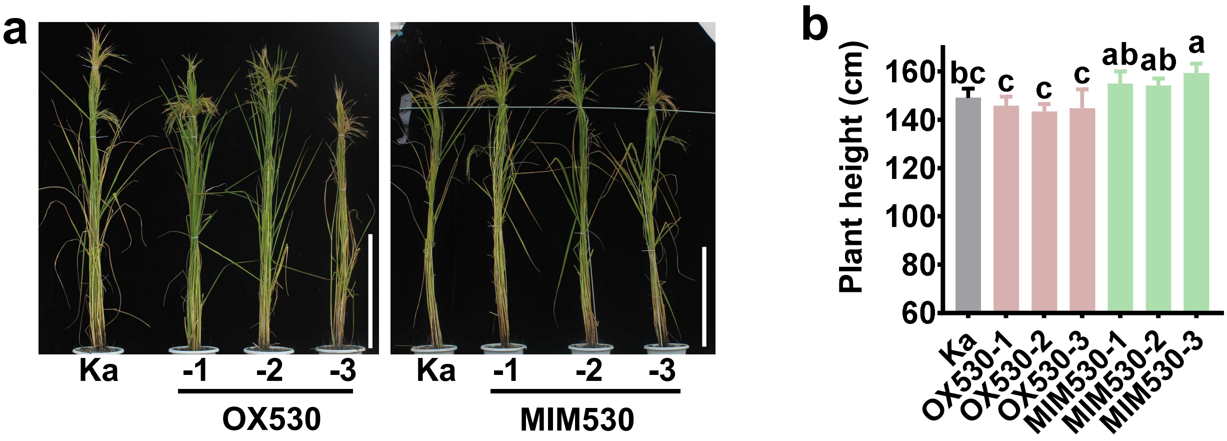

Figure S4 miR530 regulates pant height. (A) Photos of the gross morphology of the Kasalath control (Ka), OX530, and MIM530 lines planted in paddy yard in Wenjiang District, Chengdu City, Sichuan Province, China during the regular season from April to September in 2019. Scale bars=50 cm. (B) The Plant height of the indicated lines in (A). Data are shown as mean ± SD (n = 10 independent plants). Different letters above the bars indicate a significant difference ($P < 0.05$) as determined by a one-way ANOVA analysis.

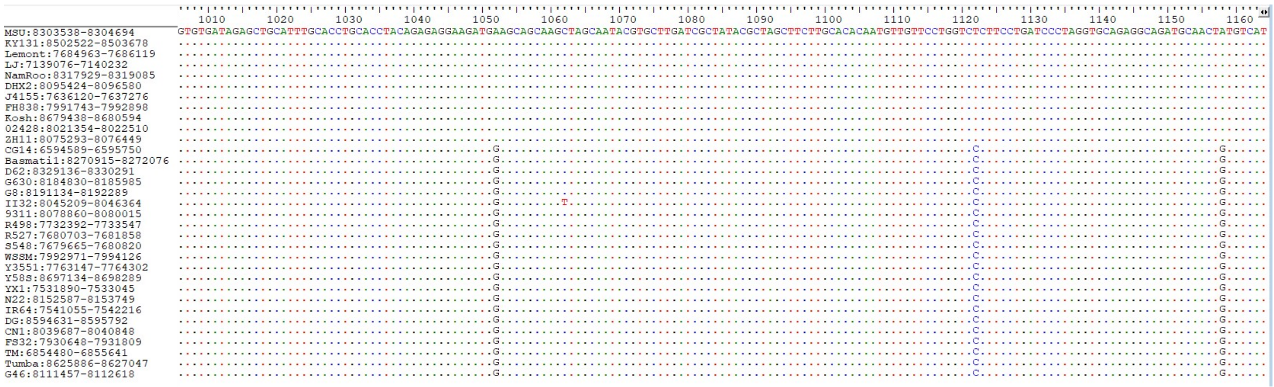

Figure S5 The sequences of miR530 precurors in the 32 *O. sativa* accessions and an ancestral species *O. glaberrima*. The sequence of Nipponbare was used as the control.
